# Annotations

**Published:** 1905-10-21

**Authors:** 


### ANNOTATIONS.

### Enteric Fever in the Army.

Ever since the actual extent of the devastation wrought by enteric fever during the South African war became generally known, the British public have been anxious that some strenuous attempt should be made by the military authorities to diminish the ravages of this scourge in the army. It is probable for this reason that the lengthy communication to *The Times* of October 17 from Surgeon-General Gallwey, principal medical officer of His Majesty's forces in India, will attract considerable attention. The author of this communication appears to be mainly concerned about denying the utility of sterilising drinking water as a measure of prevention against enteric fever. He brings forward plausible arguments and statistics to show that drinking water is only one of many channels whereby infection may be carried, and so far as India is concerned, he does not regard the boiling of water as a promising or efficient prophylactic procedure. To quote his own words, "during our close and continuous investigation of this disease in recent years it has been forcibly brought home to us all that enteric is seldom water-borne in India . . . and therefore all our schemes for sterilisation of water by boiling or otherwise have had no effect in controlling its incidence." He adds that the three years subsequent to the introduction of the universal system of boiling water in 1895 were remarkable for an exceptional increase in the prevalence of enteric fever. We must accept the evidence thus offered that enteric fever is frequently conveyed by other means than by water, but we sincerely trust that this will not be taken as a reason for ceasing to employ water sterilisation. There were 51,000 casualties from enteric fever during the war in South Africa, and no effort could be too great to forestall such prevalence in a future campaign. Prevention of infection by drinking water is at least one of the essential precautions necessary, and we hope that neither this nor any other hygienic device suitable to the circumstances will be ignored. It may be, as Surgeon-General Gallwey believes, that we can never abolish enteric fever, but it is quite certain that if the attempt be made with energy and determination, a great reduction of the disease will be brought about.

### The National Problem of Teeth.

Professor Osler has lost no time in making his influence felt as a public speaker. In his address to the successful students of the school of the Royal Dental Hospital of London, to whom he presented the prizes, the new Regius Professor of Medicine at the University of Oxford strongly impressed upon them the fact that they belong to a scientific profession, and urged them, even in these days of modern advancement, to read and diligently study John Hunter. While recognising, and insisting upon, the value of specialism, Professor Osler believes that it tends in any department to narrow-mindedness, and needs its corrective. Enlarging on the functions of a dentist, he said that one is the relief of suffering but that another and more important relates to the digestive capacity of the public, whom he divides into two great groups, the bolters and the chewers. He maintains that it is the business of dental students to endeavour to convert the overwhelming percentage of bolters into a select group of chewers. This is their mission of utility; but Professor Osler also affirms that they have a mission to beautify the race. He holds that if there is one thing more beautiful than another under heaven it is a beautiful set of teeth. To promote these missions he would have attached to every elementary school a dental surgeon to inspect the mouths of the children; and total abstainers will learn, with a shock, that he considers the question of teeth more a national problem than that of alcohol. Without discussing Professor Osler's daring comparison, we do not hesitate to endorse his opinion as to the immense importance of dental hygiene. If people generally had good teeth instead of bad, the chewers would be many and the bolters few, and a potent cause of human suffering and physical deterioration would be arrested. The tooth brush and the dentist are by no means given their proper place among the blessings of modern man.

### Cancer Research at the Middlesex Hospital.

It is probably not generally known that the Middlesex Hospital has been intimately associated with cancer and cancer research since 1792. In that year a Cancer Department was founded by one of the surgeons to the hospital—John Howard—to give special facility for studying the disease clinically and making a careful and exhaustive note on each case. At that time Howard wrote that cancer, "although extremely common is, both with regard to its natural history and cure, but imperfectly known." Now, after the lapse of 114 years, this statement is for the most part still true. During this long interval more might have been accomplished had other benefactors followed Howard's example and left funds for special research, instead of giving large donations to hospitals which exist more for the cure than the prevention of disease. It is, indeed, only during recent years that scientific investigation on a complete scale has been undertaken. The Middlesex has gradually added to its Cancer Department, and in 1900 a special wing was opened, whose wards contain in all forty-nine beds. The value of this department in the future will be greatly enhanced by work done in the new laboratories opened recently. These laboratories are situated under the Cancer Wards and a unique feature is that each member of the staff (now numbering eight) has a room to himself. How great a boon this is only those who have done research work know; this of itself is bound to increase the accuracy of all observations and results. Every encouragement such as this given to individuals who are qualified to engage in original investigation is to be welcomed. For the more competent and experienced workers there are in the quest the more likelihood there is of finding out the origin and nature of this dread disease which, in spite of many alleged "cures," is at the present time most successfully treated by the surgeon.

